# The role of long non-coding RNAs in carbohydrate and fat metabolism in the liver

**DOI:** 10.1016/j.ncrna.2023.03.003

**Published:** 2023-03-14

**Authors:** Valentin Kudriashov, Albert Sufianov, Andrey Mashkin, Aferin Beilerli, Tatiana Ilyasova, Yanchao Liang, Sergey Lyulin, Ozal Beylerli

**Affiliations:** a Gastric Cancer Center, West China Hospital of Sichuan University, China; b Educational and Scientific Institute of Neurosurgery, Рeoples’ Friendship University of Russia (RUDN University), Moscow, Russia; c Department of Neurosurgery, Sechenov First Moscow State Medical University (Sechenov University), Moscow, Russia; d Department of Obstetrics and Gynecology, Tyumen State Medical University, 54 Odesskaya Street, 625023, Tyumen, Russia; e Department of Internal Diseases, Bashkir State Medical University, Ufa, Republic of Bashkortostan, 450008, Russia; f Department of Neurosurgery, The First Affiliated Hospital of Harbin Medical University, Harbin, 150001, China; g Carmel Medical Center, Chelyabinsk, Russia

**Keywords:** lncRNAs, Liver, Carbohydrate metabolism, Fat metabolism, Signal pathways

## Abstract

The metabolism of carbohydrates and lipids (fat) in the liver is closely interconnected both in physiological conditions and in pathology. This relationship in the body is possible due to the regulation by many factors, including epigenetic ones. Histone modifications, DNA methylation, and non-coding RNAs are considered to be the main epigenetic factors. Non-coding RNAs (ncRNAs) refers to ribonucleic acid (RNA) molecules that do not code for a protein. They cover a huge number of RNA classes and perform a wide range of biological functions such as regulating gene expression, protecting the genome from exogenous DNA, and directing DNA synthesis. One such class of ncRNAs that has been extensively studied are long non-coding RNAs (lncRNAs). The important role of lncRNAs in the formation and maintenance of normal homeostasis of biological systems, as well as participation in many pathological processes, has been proven. The results of recent studies indicate the importance of lncRNAs in lipid and carbohydrate metabolism. Modifications of lncRNAs expression can lead to disruption of biological processes in tissues, including fat and protein, such as adipocyte proliferation and differentiation, inflammation, and insulin resistance. Further study of lncRNAs made it possible to partly determine the regulatory mechanisms underlying the formation of an imbalance in carbohydrate and fat metabolism individually and in their relationship, and the degree of interaction between different types of cells involved in this process. This review will focus on the function of lncRNAs and its relation to hepatic carbohydrate and fat metabolism and related diseases in order to elucidate the underlying mechanisms and prospects for studies with lncRNAs.

## 1 Introduction

About 93% of the DNA in the human genome can be transcribed into RNA, but only 2% of the RNA has a protein-coding function, and the remaining 98% of the RNA is non-coding RNAs (Non-coding RNAs, ncRNAs) [1]. According to different lengths, ncRNAs larger than 200 nt are called lncRNAs, and those shorter than 200 nts are classified as small non-coding RNAs (Small ncRNAs, sncRNAs). Thanks to the rapid development of whole-genome sequencing technology and high-throughput sequencing technology, a large number of lncRNA molecules have been discovered, and researchers have begun to re-understand the “dark matter” in the genome [2]. Related reports have shown that, unlike short-chain sncRNAs, lncRNAs can form complex high-level structures, and the long chains of nucleotides carry more biological information, with epigenetic regulation, transcriptional regulation, post-transcriptional regulation, translational regulation and Post-translational regulation and other multi-level regulatory capabilities [3]. With the deepening of research, researchers have discovered that lncRNAs plays an important role in various diseases such as cancer, metabolic diseases, and neurodegenerative diseases [4]. Therefore, lncRNAs has gradually become a research hotspot in the field of life sciences.

In recent years, the role of lncRNAs in metabolic diseases has gradually attracted attention. In diabetes, hyperlipidemia, non-alcoholic fatty liver and other diseases, the expression pattern of lncRNAs is closely related to the development of the disease [5]. As an important metabolic organ of the human body, the liver is extremely important for maintaining the homeostasis of the human body. Studying the role of lncRNAs in liver metabolism will provide an important basis and new method for the diagnosis, treatment and prognosis analysis of related metabolic diseases in the future. Therefore, this article will comprehensively review the mechanism of action of lncRNAs, its regulatory mode and related targets in liver glucose and lipid metabolism, in order to provide ideas for subsequent scholars to carry out related research.

LncRNAs was once considered to be the “dark matter” in the genome, and it did not attract the attention of researchers at the beginning of its discovery. For example, H19 and XIST were discovered in the pre-genome era, but it was not until the beginning of the 21st century that related research began. In the initial stage of research in this field, researchers generally believed that lncRNAs does not have the ability to encode proteins, so this type of RNAs is called non-coding RNAs, but with the deepening of research, researchers began to find that some lncRNAs has an “open reading frame’ (ORF), which can translate short peptides, these short peptides from lncRNA are also considered to have the ability to regulate other proteins. The sources of lncRNAs mainly include the following five types: (1) lncRNAs formed by structurally interrupted coding genes; (2) lncRNAs produced during chromatin reorganization; (3) lncRNAs formed by reverse translocation during replication; (4) lncRNAs produced in tandem with local replicons; (5) lncRNAs formed by inserting transposons into genes [6]. Similar to mRNA, the transcription of lncRNAs is also completed by RNA polymerase, which is cut to form a mature lncRNAs with a “5′ cap structure” and a “3′ poly A tail”. The same gene can form lncRNAs of different transcripts. Compared with mRNA, there are more types of lncRNA, but its expression level is much lower than that of mRNA. Current studies have found that lncRNAs exist in the genomes of multiple species, but compared with mRNAs, lncRNAs are relatively poorly conserved among different species.

The classification of lncRNAs is usually based on their function and positional relationship with nearby genes in the genome. Its types can be divided into: (1) intergenic lncRNAs, this type of lncRNAs is located in the middle of two protein-coding genes, and its transcriptional activity is also regulated by epigenetics. (2) Intron lncRNAs, this type of lncRNAs comes from inside the DNA sequence of a gene, and is identical to the partial sequence of the sense strand or antisense strand of the gene intron. Intronic lncRNAs are less conserved than intergenic lncRNAs. (3) Sense lncRNAs. (4) Antisense lncRNAs. (3) and (4) These two types of lncRNAs are transcribed from exons of protein-coding genes, which can either include the entire protein-coding gene or partially overlap with the gene. (5) Enhancer lncRNAs, this type of lncRNA is transcribed from the promoter region of protein-coding genes [7].

**Fig. 1:**
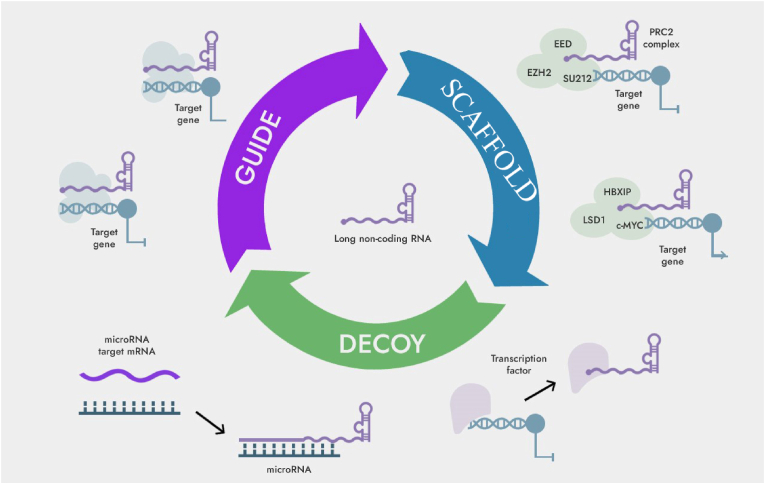
Functions of long non-coding RNAs.

Different from microRNAs (miRNAs), lncRNAs have complex secondary and tertiary structures, which can act as “scaffolds” in the process of forming protein complexes, realizing the crossover and integration of information between different signaling pathways, or Binding to individual proteins regulates protein function and/or stability at the post-translational level (Fig. 1).

At present, with the development of high-throughput sequencing technology, it is also possible to detect the secondary structure of lncRNAs. The application of RNA structure probe technology has revealed the secondary structure of some important lncRNAs. For example, the antisense RNA (HOX trans antisense RNA, HOTAIR) transcribed from the HOX locus has four domains, consisting of 36 helices, consists of 38 cones, 34 internal loops and 19 junctional regions, evolutionarily conserved; nuclear-enriched autosomal transcript 1 (NEAT1) forms 4 domains, and NEAT1_L (one of NEAT1 There is RNA-RNA interaction between the 5′ end and the 3′ end of the subtype) [8]. The above research results show that lncRNA is usually folded into multiple domains, and different domains play an important role in its normal function. The structural biology research of lncRNAs will contribute to the in-depth exploration of lncRNAs mechanism of action and regulatory process.

LncRNAs binds to genomic DNA, usually by binding to *cis*-acting elements on the gene promoter to regulate gene transcription. This type of lncRNAs usually repeats the sequence at the 5′-end boundary of the gene, and basically does not contain the exon sequence of the gene. On the one hand, lncRNAs can combine with the promoter region of DNA to form a DNA-RNA triple-strand hybrid fragment, preventing transcription factors from starting the transcription process, thereby inhibiting gene expression; “connection tool”, which recruits protein complexes with transcriptional regulatory functions to the promoter segment of DNA to initiate chromatin remodeling, activate or inhibit transcription initiation, and perform epigenetic regulation through histone modification [9].

Based on the nucleic acid structure of lncRNAs itself, the combination between lncRNAs and mRNA and miRNA becomes relatively easy. By binding to mRNA, lncRNAs can regulate mRNA splicing, intracellular distribution and stability. The combination between lncRNAs and miRNA is usually more like the adsorption of “sponge”, which reduces the binding between miRNA and target gene and reduces the inhibitory effect of miRNA on its target gene [10].

In addition, as mentioned above, some lncRNAs have shorter ORFs and can translate short peptides. For example, LINC00961, as a lncRNAs, can translate a polypeptide SPAR consisting of 90 amino acids, which has the function of inhibiting muscle regeneration [11].

All in all, lncRNAs are involved in the regulation of gene expression at various levels, including epigenetic modification, transcriptional regulation, RNA splicing, nuclear shuttling, post-transcriptional level, translational level, and post-translational level, basically running through the currently known. It is worth noting that there are still many unknown regulatory mechanisms that we still do not understand, and the research on lncRNA needs to be further in-depth.

As one of the most important organs in the human body, the liver plays an important role in maintaining the energy metabolism of the body. In terms of glucose metabolism, the liver is involved in processes such as glycogen breakdown, synthesis, and gluconeogenesis to maintain blood glucose levels. In terms of lipid metabolism, the liver is involved in the synthesis and transport of lipids. Since the liver is not a lipid storage organ in the body, the triglycerides, phospholipids, cholesterol and apolipoproteins it synthesizes form very low-density lipoproteins, which are transported through the blood to the body organs or stored in adipose tissue. Therefore, the liver plays an important role in maintaining the homeostasis of glucose and lipid metabolism in the body.

The liver plays an important role in maintaining lipid homeostasis in the human body. Non-alcoholic fatty liver disease (NAFLD), non-alcoholic hepatitis, and even cirrhosis and liver cancer can develop due to liver lipid disorders. However, mild fatty liver disease usually doesn't show any symptoms and is difficult to diagnose clinically. The differential expression of lncRNA between normal individuals and those with abnormal liver lipid metabolism shows promising potential for the future development of lncRNAs as a biomarker or target for medical detection of this type of disease. (Table 1).

**Table 1:** Functional lncRNAs involved in hepatic lipid metabolism.

Name	Target	Function	Ref.
MALAT	SREBP1C	SREBP1C↑, ACC1↑, FAS↑	[36]
ARSR	Akt/SREBP-2/HMGCR	Akt↓, SREBP-2↑, HMGCR↑, TC↑	[34]
uc.372	miR-195	miR-195↑, AGO↓, ACC↓, FAS↓	[12]
H19	MLXLPL,mTOR	MLXLPL↑, mTOR↑, FASN↑, Acaca↑, Scd1↑	[25]
	miR-130a	miR-130a↓, PPARγ↑, FASN↑, ACC1↑, SCD↑	[24]
	PTBP1	PTBP1↑, SREBP1C↑, FASN↑	[23]
GM16551	Srebp1c	FAS↓, SCD↓, serumTG↓	[35]
LSTR	TDP43	Cyp8b↑, MCA/CA↓, Apoc2↓	[31]
AT102202	HMGCR	HMGCR↓	[37]
SRA	FoxO1	FoxO1↓, ATGL↓, SREBP1C↓, FASN↓	[22]
NEAT1	mTOR/S6K1	ACC↑, FAS↑	[13]
	miR-124-3p	miR-124-3p↓, ATGL↑, DAG+FFA↑, lipolysis↑	[16]
	miR-146b-5p	miR-146b-5p↓, Rock↓, Ampk7↑, steatosis↓	[15]
	miR-140	miR-140↑, AMPK/SREBP1c↑, NAFLD↑	[14]
APOA4-AS	APOA4	HUR↑, APOA4↑, plasm TC↑, TG↑	[27]
HC	hnRNPA2/B1	HC↑, Cyp7a1↓, Abca1↓	[30]
	miR-130b-3p	HC↑, PPARγ↑, TG↓	[31]
MEG3	PTBP1	PTBP1↓, SHP↓, Cyp7a/8b↑, bile acid↑	[28]
	miR21	LRP6↑, AKT↑, p-mTOR↑, TG↑	[29]
Lexis	RALY	Srebf2↓, Pcsk9↓, cholesterol↓	[21]
LASER	LSD	LSD↑, HNF-1α↓, PCSK9↑, cholesterol↑	[33]
HR 1	PDK1/AKT/FoxO1	PDK↑, AKT↑, FoxO1↑, SREBP1C↑, TG↑	[18]
Blnc1	Srebp1c	Srebp1c↑, Fasn↑, AST↑, ALT↑	[20]
LincIRS2	MAFG	Acox1↓, Cpt1a↓	[48]
KDM5D-4	PLIN2	PLIN2↓, Lipid droplet↓	[32]
SHGL	hnRNPA1	hnRNPA1↓, CALM↑, mTOR/SREBP-1C↓, liver TG↓	[46]

Uc372 expression was found to increase in the liver tissues of NAFLD patients, HFD and db/db mice, and it is primarily distributed in the nucleus of the liver. The mature microRNA uc372 binds to primary transcripts of miR-195 and miR-4668, preventing their splicing modification, which leads to the activation of lipid-synthesis associated genes, including Srebp1, Acc, Fas, and CD36 protein. This ultimately results in increased liver lipid activity [12].

The research on the regulation of liver lipid metabolism by LncNEAT1 first began in 2017. Wang found that NEAT1 promoted the expression of Acc and Fas in hepatocytes by activating the Mammalian target of rapamycin (mTOR)/S6K1 signaling pathway. On the contrary, the use of lentivirus to interfere with NEAT1 can alleviate the non-alcoholic fatty liver disease caused by HFD [13]. Other studies have shown that there is an interaction between NEAT1 and miR-140. Inhibition of NEAT1 in vitro can down-regulate the expression of miR-140, on the contrary, inhibition of miR-140 can also reduce the expression of NEAT1, but any interference with either can increase the phosphorylation of AMP activated protein kinase (AMPK) reduce the expression levels of Srebp1, Fas and Acc genes, and play a role in lowering lipids [14]. Chen et al. found that NEAT1 can also bind to miR-146a-5p and release the inhibitory effect of miR-146-5p on Rho-associated protein kinase 1 (Rock1) [15]. As the upstream gene of AMPK, Rock1 can inhibit its activity and promote the body's lipid synthesis. In addition to participating in lipid synthesis and metabolism in the liver, NEAT1 can also regulate abnormally altered lipid catabolism in liver cancer. The study found that the expression of NEAT1 was significantly increased in HCC cells. NEAT1 weakens the inhibitory effect of miR-124-3p on Atgl by combining with miR-124-3p, indirectly promotes the expression of Atgl, and enhances the lipid hydrolysis process of cells [16].

LncHR1 (HCV regulated 1) is a lncRNA that can be activated by hepatitis C virus (HCV). However, in the process of HCV promoting liver lipid synthesis, HR1 did not play a role in mediating the development of this process, but reduced the body's lipid accumulation by inhibiting the expression of SREBP-1C [17]. Furthermore, in vivo and in vitro experiments showed that HR1 can alleviate the excessive lipid accumulation caused by HFD. Li et al. further found that the inhibitory effect of HR1 on SREBP-1C is mediated by the regulation of pyruvate dehydrogenase kinase 1 (Pyruvate dehydrogenase kinase 1, PDK1)/AKT/FoxO1 pathway) [18]. The results showed that HR1 inhibited the phosphorylation of PDK1, weakened the phosphorylation and activation of AKT by PDK1, and then affected the nuclear and cytoplasmic distribution of FoxO1, increasing the nuclear import of FoxO1, reducing the expression of SREBP-1C, and alleviating liver lipid accumulation.

The expression of LncRNA liver-specific triglyceride regulator (LncLSTR) was significantly reduced in the liver tissue of mice treated with 24 h starvation, and its expression was quickly restored with re-feeding. In vivo experiments showed that liver-specific knockout of LSTR enhanced the clearance rate of TG in the blood of mice and decreased the content of TG in the blood of mice. Further studies found that LSTR combined with nucleic acid binding protein TDP-43, weakening the inhibitory effect of TDP-43 on Cyp8b1. After LSTR is inhibited, the expression of Cyp8b1 decreases, which changes the ratio of bile acid to bile acid in the body, activates the bile acid receptor FXR, and promotes the expression of Apoc2. Increased expression of Apoc2 enhances the activity of lipoprotein lipolytic enzymes, thereby increasing the clearance rate of TG in the blood [19].

Brown fat-enriched long noncoding RNA 1 (Brown fat-enriched lncRNA 1, LncBlnc1) was first discovered in adipose tissue, which can regulate the differentiation and thermogenesis of brown adipose tissue. With the deepening of the research, the researchers found that Blnc1 was expressed in a higher abundance in the liver. The expression of Blnc1 was significantly increased in the liver tissues of HFD mice, ob/ob mice and db/db mice. Studies have found that Blnc1 promotes the assembly of the liver X receptor (Liver X receptors, LXR) transcription complex by combining with EDF1. Interfering with Blnc1 weakens the binding between EDF1 and LXR and the recruitment of LXR on the SREBP-1C promoter, reducing the expression of genes related to lipid synthesis [20].

LXR is an important regulator of cholesterol metabolism in the body, which can promote cholesterol efflux and esterification and inhibit cholesterol absorption. Sallam et al. treated primary hepatocytes with the LXR agonist GW3965 and then performed a transcriptomic analysis to weed out the most overexpressing lncRNA and named it as liver LXR-expressed lncRNA (liver LXR-expressed lncRNA, sequence, LeXis), and found that in addition to being involved in the above regulatory pathways, LXR can also inhibit cholesterol synthesis in the body [21]. Studies have shown that LeXis is predominantly localized to the nucleus, induced and activated by LXR, and coupled to the heterogeneous ribonucleoprotein RALY. RALY acts as a transcriptional coactivator, promoting the expression of genes associated with cholesterol biosynthesis. The combination of LeXis and RALY inhibits the transcriptional regulation function of RALY, thereby inhibiting cholesterol synthesis in the body.

Steroid receptor RNA activator LncRNA (steroid receptor activator RNA, LncSRA) is a lncRNA involved in various physiological functions, SRA as a RNA transcription coactivator can simultaneously enhance gene expression mediated by steroid and non-steroid nuclear receptors. Plays a role in the regulation of adipose tissue differentiation and glucose uptake by adipose tissue. Studies have shown that in liver tissue, SRA reduces ATGL expression by increasing the level of FoxO1 phosphorylation induced by insulin signaling. The level of FoxO1 phosphorylation usually determines the biological activity and nucleoplasmic distribution of FoxO1. In the absence of insulin, SRA overexpression increased the level of FoxO1 phosphorylation, but significantly increased FoxO1 import into the nucleus. At the same time, an experiment with the luciferase reporter gene showed that increased nuclear import of FoxO1 caused by SRA overexpression does not increase the Atgl promoter activity, and the specific molecular mechanism is still unclear [22].

In addition to being involved in the maintenance of hepatic glucose homeostasis, H19 also plays an important role in hepatic lipid metabolism. Studies have shown that H19 expression is significantly upregulated in the livers of mice fed a high fat/sucrose (HFHS) diet. Accordingly, intervention in H19 may alleviate lipid accumulation caused by the HFHS diet. Further studies showed that H19 in the liver binds to the hnRNP family member PTBP1, on the one hand, promotes binding between PTBP1 and Srebp-1c mRNA, improves the stability of Srebp-1c mRNA, on the other hand, promotes interaction between PTBP1 and SREBP-1C protein precursors increases the penetration of SREBP-1C into the nucleus, simultaneously enhancing the transcriptional activity of SREBP-1C, thereby promoting lipid synthesis in the liver [23]. The peroxisome proliferator-activated receptor γ (PPARγ) is a transcription factor highly expressed in adipose tissue and plays an important role in the regulation of lipid metabolism. It has been reported that PPARγ is induced in the liver of NAFLD patients, leading to accumulation of lipids in the liver. Liu et al. found that H19 can bind to miR-130a and counteract miR-130a′s inhibitory effect on PPARγ, thereby promoting hepatic lipid synthesis [24]. Additional studies have shown that, in addition to regulating the above transcription factors, H19 may also promote hepatic lipid synthesis by promoting the expression of carbohydrate response element binding protein (Chrebp) and activating the mTOR signaling pathway. From the current study, it is easy to see that H19 acts as a “sensor of lipid synthesis” and is induced into expression when the amount of free fatty acids in the blood increases. It promotes lipid synthesis in the liver at different levels of metabolic regulation and signaling pathways, reduces the content of free fatty acids in blood lipids [25].

LncAPOA1-AS was found to regulate the expression of liver apolipoprotein A1 (Apolipoprotein A1, Apoa1). APOA1-AS, as the antisense sequence of Apoa1 DNA segment, overlaps with the fourth exon of Apoa1. Studies have shown that interfering with APOA1-AS can weaken the binding of protein demethylase (Lysine (K)-specific demethylase1A, LSD1) at the H3K4 site and the binding of polycomb repressive complex 2 at the H3K27 site on the Apoa1 promoter, respectively. Combined, it can increase the level of monomethylated lysine 4 on histone H3 (H3K4me) while reducing the level of H3K27me3, and promote the expression of APOA1 [26]. Subsequently, APOA4-AS was also found to regulate lipoproteins. Similar to APOA1-AS, APOA4-AS is an antisense sequence that overlaps with the third exon and 3′ non-coding region of Apoa4. Differently, APOA4-AS forms a complex with the mRNA-binding protein HuR, which binds to and enhances the stability of Apoa4 mRNA. The expression of APOA4-AS and Apoa4 in the liver of human fatty liver patients and ob/ob mice showed a tendency to increase, and inhibiting APOA4-AS could significantly reduce the content of TG and TC in the blood of ob/ob mice [27].

LncMEG3 can also bind PTBP1 and recruit PTBP1 to the small heterodimeric partner (small heterodimer partner, SHP) mRNA by combining with PTBP1 and promote the degradation of Shp mRNA. MEG3 does not directly mediate mRNA degradation in the entire process. Instead of degradation, it plays the role of “guide” and “stent”. SHP has been shown to be a bile acid synthesis repressor molecule, and inhibition of SHP induces the expression of cholesterol 7a-hydroxylase (cholesterol 7-alpha-hydroxylase, Cyp7a1) and cholesterol-12α-hydroxylase (sterol-12a-hydroxylase, Cyb8b1). activate the metabolism of bile acids. In fact, in liver tissue, there is a close relationship between bile acid metabolism and lipid metabolism, for example, FXR can be activated by bile acid metabolites and plays an important role in lipid metabolism [28]. Huang et al. used a non-alcoholic fatty liver mouse model for research and found that MEG3 can act as a cRNA to bind miR-21 and attenuate the effect of miR-21 on LDL-related protein 6 [29]. Protein related to the low density lipoprotein receptor 6) 6. Inhibition of Lrp6 mRNA. Activated LRP6 inhibits AKT/mTOR signaling and reduces hepatic lipid synthesis.

Regarding lncRNA regulation of cholesterol metabolism, Lan et al. found that lncHC is expressed in the liver of HFD mice and is involved in hepatic cholesterol metabolism [30]. When liver cholesterol levels rise, LXRα is activated to stimulate the expression of the transcription factor CCAAT-enhancer-binding protein beta (CCAAT/enhancer-binding protein beta, C/EBPβ). Subsequently, HC is activated by C/EBPβ, binds to hnRNPA2B1 and recruits it to the mRNA of the Cyp7a1 and Abca1 HC target genes, reduces the stability of the target gene mRNA, and inhibits bile acid metabolism and high density lipoprotein synthesis. The author then pointed out in another article that HA can not only regulate bile acid metabolism, but also regulate lipid synthesis in the liver. Experiments have shown that HC promotes miR-130b-3p expression, inhibits miR-130b-3p PPARγ target gene expression, reduces expression of fatty acid absorption and lipid synthesis-related genes in the liver, and reduces lipid accumulation in liver [31].

The LncKDM5D-4 locus is located on the Y chromosome. Interfering with KDM5D-4 in HepG2 cells can increase the expression of lipid droplet coating protein 2 and promote the formation of lipid droplets in the liver [32].

LncRNA lipid-associated single nucleotide polymorphism region (Lipid associated single nucleotide polymorphism region, LncLASER) can be activated by LXR, the activated LASER can directly bind to LSD1, H3K4me demethylation occurs on the Hnf1α promoter, activation Expression of HNF-1α. HNF-1α then promotes the expression of its downstream gene, Proprotein convertase subtilisin/kexin type 9 (Pcsk9). Elevation of Pcsk9 weakens the liver's ability to remove cholesterol from the blood, resulting in hypercholesterolemia [33].

LncARSR was found to be elevated in the liver of hypercholesterolemic patients. Animal experiments have shown that overexpression of ARSR can increase the expression of cholesterol-related genes, such as hydroxymethylglutaryl CoA reductase (Hmgcr), Hmgcs, squalene synthase (Squalene synthase, Sqs). Further studies have shown that ARSR increases the expression of Srebp-2 and promotes the synthesis of cholesterol by activating the PI3K/Akt signaling pathway [34].

Gm16551 was found to be downregulated in the liver of starving mice and restored upon refeeding. Gm16551 expression was upregulated by SREBP1C, and Gm16551 expression was negatively correlated with genes associated with lipid synthesis. Experiments have shown that intervention in Gm16551 in mice increases the expression of Acly, FasS, and Scd1; when Srebp1c is knocked down, the levels of genes associated with lipid synthesis are suppressed based on intervention in Gm16551; with the synthesis of lipids activated by SREBP1c. Therefore, Gm16551 is considered to be a SREBP1c negative feedback element that promotes hepatic lipid synthesis, which is expressed under the control of SREBP1c and inhibits SREBP1c-activated lipid synthesis [35].

LncMALAT1 expression was found to be significantly increased in liver tissues of ob/ob mice as well as HepG2 cells and primary hepatocytes treated with palmitic acid. Conversely, inhibition of MALAT1 expression in vivo and in vitro resulted in hepatic lipid accumulation. Studies have found that MALAT1 can combine with SREBP1C, reduce the ubiquitination and degradation of SREBP1c, improve the stability of SREBP1c, and promote lipid accumulation in the liver [36].

Epigallocatechin-3-gallate (EGCG), the main component of green tea extract, can lower blood cholesterol levels. The study found that treatment of HepG2 cells with EGCG can activate the expression of lncRNAAT102202. AT102202 consists of 303 amino acids, and its sequence is 100% similar to the sequence of the 4th–6th exon on the HmgcrDNA sequence of the gene related to cholesterol synthesis in the liver. At the same time, interference with AT102202 in HepG2 cells resulted in a decrease in HMGCR expression [37].

LncRNA directly or indirectly regulates the rate-limiting enzymes of lipid metabolism (ACC, HMGCR, SREBP1c, PPARγ, CYP7a1, etc.), and changes in the above genes at the level of transcription or protein can most intuitively reflect the state of lipid metabolism in the liver, which became the subject of research for scientists.

### 1.1 Carbohydrate metabolism

The liver plays an important role in maintaining the body's blood sugar concentration. When fasting, blood sugar is low, and the liver promotes glycogenolysis and gluconeogenesis to increase blood sugar concentration, after eating, blood sugar is high, and the liver can promote glycogen synthesis, inhibit glycogenolysis and gluconeogenesis to lower blood sugar levels. In recent years, lncRNAs has been found to play an important role in hepatic glucose metabolism (Table 2).

**Table 2:** Functional lncRNAs involved in hepatic glucose metabolism.

Name	Function	Ref.
MEG3	miR-302a-3p↓, G6pase↑, CRTC2↑, Pck1↑ miR214↓, G6pase↑, FoxO1↑, ATF4↑, IR↑, Pck1↑ miR-185-5p↓, Egr2↑, IR↑	[[[42], [43], [44]]]
Gomafu	miR-139-5p↓, G6pase↑, FoxO1↑, IR↑, Pck1↑	[[45]]
SHGL	hnRNPA1↓, CALM↑, Pck1↓, (PI3K)/Akt/FoxO1↑, G6pase↓	[[46]]
LGR	hnRNPL↓, G6pase↑, GCK↓, Pck1↑	[[47]]
LincIRS2	Pck1↓, G6pc↓, FoxO1↓	[[48]]
Gm10768	miR214↓, Pepck↑, ATF4↑, G6pase↑	[[49]]

As a multifunctional long non-coding RNAs, H19 can function in both the nucleus and the cytoplasm. Goyal et al. detected 3 lncRNAs with significantly decreased expression levels when sequencing the transcriptome of db/db mice, among which the change of H19 is the most drastic [38]. Inhibition of H19 in hepatoma cell HepG2 and primary hepatocytes can promote gluconeogenesis genes Glucose-6-phosphatase (G6pc), phosphoenolpyruvate carboxykinase (Recombinant Phosphoenolpyruvate Carboxykinase 1, Pck) expression and increase cellular glucose production. In vivo experiments showed that inhibiting the expression of H19 in mouse liver by siRNAs would cause hyperglycemia and hyperinsulinemia in mice [39]. Further studies have found that inhibiting the expression of H19 in HepG2 can increase the expression of Forkhead transcription factor O1 (FoxO1) and affect its subcellular translocation, prompting more FoxO1 to translocate into the nucleus, and regulate gluconeogenesis. expression of related genes. However, Zhang et al. found that under starvation conditions, the expression level of H19 in mouse tissues was significantly increased; in addition, in high-fat diet (High-fat diet, HFD) mice and type Ⅱ diabetes patients; in the liver tissues of HepG2 cells, the expression of H19 showed an upward trend; in vitro experiments showed that after interfering with the expression of H19 in HepG2 cells, the glucose output of cells decreased [40]. This result is completely opposite to the above research results. Corresponding in vivo experimental results showed that liver-specific overexpression of H19 could cause insulin resistance in mice and affect glucose homeostasis in vivo. Researchers found that this phenomenon is related to the regulation of gene methylation by H19 [40]. H19 can bind to S-adenosyl homocysteine hydrolase and inhibit the activity of the enzyme, resulting in high levels of S-adenosine. Accumulation of cysteine, which in turn inhibits DNA methyltransferases. In mouse liver tissue, the increase of H19 can reduce the methylation of the CpG island on the hepatocyte nuclear factor-4α (Hepatocyte nuclear factor 1A, Hnf4α) promoter and promote its expression. Subsequently, the activated HNF4a starts the body's gluconeogenesis process. The above two studies obtained completely opposite experimental results in different animal models and the same cell model, and the reason for this contradiction is still unclear. Regarding the current data discussion, the difference between the two studies is that Zhang et al. used starvation mouse models and HFD mouse models to demonstrate under physiological and pathological conditions, while Goyal et al. In the experiment, db/db mice were used for research, and the change trend of H19 expression in the experimental results is the same as that of H19 in diabetic patients, which has better reference value [39,40].

The study found that the expression of Maternally expressed gene 3 (MEG3) was significantly increased in the liver tissues of HFD and ob/ob mice and in primary liver cells stimulated with palmitic acid, oleic acid and linoleic acid. In response to this finding, the researchers studied from an epigenetic perspective and found that the expression of HDAC1 and HDAC3, members of the HDAC family of histone deacetylases, was significantly reduced in palmitic acid-stimulated primary liver cells, while inhibition using HDAC Trichostatin A dose-dependently increased the expression of MEG3. However, overexpression of MEG3 in primary hepatocytes using adenovirus promoted the expression of FoxO1 and its downstream genes G6pc and Pck. A series of experiments showed that MEG3 in hepatocytes is activated by lipid accumulation and promotes the expression of gluconeogenesis genes. Inhibition of MEG3 expression in HFD mice and ob/ob mice alleviated impaired glucose tolerance in obese mice, although body weight was not altered [41]. Another study showed that glucagon can induce the expression of MEG3 in hepatocytes. Under the stimulation of glucagon, MEG3 is activated by cyclic adenosine monophosphate response element binding protein (cAMP-response element binding protein, CREB), and performs the function of endogenous competing RNA (Competing endogenous RNA, ceRNA), and miR-302a -3p binds to and indirectly activates the expression of Crtc2, a family member of its target gene CREB-regulated transcription coactivator (Crtc), thereby promoting the transcriptional co-activator Pgc-1α related to hepatic gluconeogenesis and its downstream glucose Expression of xenobiotic-related genes G6pc and Pck [42]. In addition, MEG3, as a ceRNA, can also bind to miR-214, reduce the inhibitory effect of miR-214 on activating transcription factor 4 (Activating transcription factor 4, Atf4), and increase the transcription of Atf4. As a transcriptional coactivator, Atf4 binds to FoxO1 and increases the transcriptional activity of FoxO1, thereby activating the body's gluconeogenesis [43]. With the in-depth research on MEG3, Chen et al. found in the latest study that, in addition to regulating the gluconeogenesis process, MEG3 can also regulate the insulin sensitivity of liver tissue and affect insulin-mediated hepatic Cellular uptake of glucose [44]. In addition, MEG3 can bind miR-185-5p and weaken the inhibitory effect of miR-185-5p on early growth response proteins-2 (Egr2) mRNA. Insulin resistance in the liver is reduced, and the absorption of glucose by liver cells is reduced.

Similar to MEG3, Gomafu is also a long non-coding RNA whose expression is significantly increased in starved mice, HFD mice and db/db mice. Studies have found that in obese mice, the combination of NF-κB family member p65 and Gomafu promoter region increases, which promotes the transcription of Gomafu, resulting in increased expression of Gomafu in liver tissue of obese mice. Conversely, inhibiting the expression of Gomafu in the liver of obese mice can reduce the progress of gluconeogenesis and improve insulin sensitivity in mice. With the same function as most lncRNAs, Gomafu also acts as a ceRNA in the regulation of gluconeogenesis, and relieves the inhibitory effect of miR-139 on FoxO1 by combining with miR-139 [45].

LncRNAs suppressor of hepatic gluconeogenesis and lipogenesis (LncRNA suppressor of hepatic gluconeogenesis and lipogenesis, LnsSHGL) is a highly expressed lncRNAs in liver tissue [46]. Expression was decreased in liver tissues of mice (HFD and db/db mice) and patients with non-alcoholic fatty liver disease. Further experiments showed that lncSHGL can recruit nuclear heterogeneous protein A1 (Heterogeneous nuclear ribonucleoprotein A1, hnRNPA1), accelerate the translation of calmodulin (Calmodulin, CaM), thereby increasing the protein expression of CaM. As CaM protein levels rise, the phosphatidylinositol 3-kinase (PI3K)/Akt pathway is activated, inhibiting the body's gluconeogenesis process.

The expression abundance of LncRNA glucokinase repressor (Liver GCK repressor, LncLGR) in the whole body of mice is low, but LGR in the liver is significantly activated under starvation conditions and returns to normal expression levels with refeeding after starvation. Further studies have found that LGR is mainly distributed in the nucleus of liver cells. After being stimulated by nutritional signals, LGR in the nucleus binds to hnRNPL and recruits it to the promoter of glucokinase (GCK) to inhibit the transcription of Gck and reduce the level of glycolysis in the body and promote the accumulation of glycogen [47].

LincIRS2 is an lncRNA localized in the nucleus. Studies have shown that its expression level can actively respond to changes in external nutritional signals. The expression of lincIRS2 in the liver tissue of mice with diet-induced obesity (DIO) decreased significantly, while in wild-type mice, the expression of lincIRS2 increased with small Fasting and re-feeding treatments showed a change that first increased and then decreased. Further studies found that the transcription factor small Maf protein MAFG inhibits its expression by binding to the lincIRS2 promoter. Under starvation conditions, MAFG is recruited by nuclear factor erythroid-2-related factor (NRF1/NFE2L1) and forms a heterodimer with it. Thereby reducing the enrichment of MAFG in the lincIRS2 promoter region and promoting the transcription of lincIRS2. In contrast, CRISPR/Cas9-and RNAi-mediated loss of lincIRS2 resulted in elevated blood glucose, insulin resistance, and abnormal glucose output in lean mice. In contrast, when lincIRS2 was activated, the mice maintained healthy blood sugar levels despite gaining weight. In summary, the MAFG-lncRNA axis plays a role in regulating hepatic glucose metabolism under physiological and pathological conditions [48].

The author's research group found that the expression of GM10768 in the liver was significantly increased after the mice were fasted for 16 h, and returned to the normal expression level when the mice were fed again. In addition, the study found that the expression of GM10768 was increased in the liver tissue of db/db mice. Further studies found that overexpression of GM10768 in primary hepatocytes increased the glucose output of the cells. Conversely, interference with GM10768 in db/db mice inhibited hepatic gluconeogenesis and alleviated hyperglycemia in mice. Experimental results show that, like MEG3, GM10768 functions as a ceRNA in liver tissue, binds to and inhibits miR-214, and promotes liver gluconeogenesis in mice [49].

Glucose levels are strictly controlled by multiple mechanisms in mammals to meet systemic energy demands, and current studies have revealed that lncRNAs play an important role in liver glucose metabolism. Table 2 lists the lncRNAs related to liver glucose metabolism mentioned in the article. It is not difficult to find that FoxO1, as a key factor in the regulatory network of islet β cells, can promote hepatic glucose production, and is also the target gene of miRNAs that are more studied in glucose metabolism. And lncRNAs can act as RNA sponge in vivo, absorb miRNAs, and further affect the role of miRNA target genes. In addition, lncRNAs can combine with RNA-binding proteins to form a functional axis and act on genes related to glucose metabolism, thereby regulating glycogen synthesis or decomposition.

## 2 Conclusion

In conclusion, lncRNAs regulate different biological processes of cell metabolism and have significant differential expression in liver metabolic diseases. Although lncRNAs were initially considered non-functional, lncRNAs genes become lncRNAs after transcription and splicing, and play specific functions in different cellular regulatory pathways [[50], [51], [52], [53]].

Binding prevents it from interacting with target gene mRNA. The expression patterns of lncRNAs in tissues coincide with different processes of diseases. Due to this specificity, lncRNAs have gradually become more potential targets in the treatment of glycolipid disorders. Although these studies are still in their infancy, researchers are beginning to take advantage of these properties of lncRNAs and apply them in clinical work. Studies have shown that lncRNAs have been used as biomarkers in various cancers. However, the current research on lncRNAs is still in its infancy, and there are still many issues that need to be further explored: (1) The research on the secondary structure, function and molecular mechanism of lncRNAs is not enough. (2) The role of lncRNAs in tumors is still the most studied, but the research on liver energy metabolism is not comprehensive. (3) Most of the current research level on lncRNAs is only in the experimental stage, and there is still a long way to go before clinical application. However, lncRNAs has great significance in liver metabolic diseases, which means that lncRNAs will play an important role in the diagnosis, treatment and prognosis of such diseases.

## Funding

This work was supported by the Bashkir State Medical University Strategic Academic Leadership Program (PRIORITY-2030).

## Author contributions

Valentin Kudriashov and Albert Sufianov conceptualized and designed the study. All authors have participated in the acquisition, analysis and interpretation of the data. Aferin Beilerli and Andrei Mashkin has drafted the manuscript. Andrey Kostin, Tatiana Ilyasova, Yanchao Liang contributed to the critical revisions of the manuscript. Ozal Beylerli supervised the research. All authors agreed on the journal to which the article would be submitted, gave the final approval for the version to be published, and agreed to be accountable for all aspects of the work.

## Declaration of competing interest

The authors declare that no conflicts of interest exist.
